# An overview of Estonian woodlice (Isopoda, Oniscidea)

**DOI:** 10.3897/zookeys.1067.68105

**Published:** 2021-10-29

**Authors:** Kaarel Sammet, Getriin Orgusaar, Mari Ivask, Olavi Kurina

**Affiliations:** 1 Estonian University of Life Sciences, Institute of Agriculture and Environmental Sciences, Kreutzwaldi st 5-D, 51006 Tartu, Estonia Estonian University of Life Sciences Tartu Estonia; 2 University of Tartu, Faculty of Science and Technology, Vanemuise 46, 51014 Tartu, Estonia University of Tartu Tartu Estonia; 3 Tallinn University of Technology, School of Engineering, Tartu College, Puiestee 78, 51008 Tartu, Estonia Tallinn University of Technology Tartu Estonia

**Keywords:** Estonia, Oniscidea, range shifts, soil arthropods

## Abstract

An overview of the Estonian terrestrial isopod fauna is given, based on literature data and material collected from 1984 to 2021. The identified material consisted of 10915 specimens belonging to 14 species and collected from 172 localities throughout Estonia. In combination with previous data from the literature data, there are now reliable records of 16 species of woodlice from Estonia. Two species, viz. *Platyarthrushoffmannseggii* Brandt, 1833 and *Hyloniscusriparius* (C. Koch, 1838), are new for the fauna. The latter has probably colonised Estonia recently and range expansions have been reported elsewhere. The data on *Philosciamuscorum* (Scopoli, 1763) are dubious, and this species is currently excluded from the Estonian list.

## Introduction

The knowledge on Estonian terrestrial isopods is scattered in various publications, without a modern overview of the fauna. Some publications are in Estonian and may be thus inaccessible to the wider audience.

Data on this group were first given by J. B. Fischer, who mentioned the presence of *Oniscusasellus* Linnaeus in Livonia (Fischer 1778: 167), an earlier administrative division, which covered the southern part of present-day Estonia and northern Latvia. The identity of the abovementioned species is unclear, as most of European species were yet to be described. At the beginning of 20^th^ century, W. Herold collected material in many places in Estonia and Latvia, published the results in several works (Herold 1927, 1928, 1930), and provided the first reliable overview of the fauna, which included 13 Estonian species. Later, Estonian entomologist J. Vilbaste published new records in three local faunistic studies (Vilbaste 1970; Vilbaste et al. 1985; Vilbaste and Vilbaste 1993) and K. Remm added one record (Remm 1988).

A lot of unidentified material from various research projects and fieldwork made over many years (1984–2020) has been stored in the entomological collection of Estonian University of Life Sciences (including the zoological collections of the former Institute of Zoology and Botany of the Estonian Academy of Sciences) and Tallinn University of Technology (TalTech) soil biology laboratory. Based on these materials and literature records, an account of the current knowledge is given below.

## Material and methods

**Figure 1.**
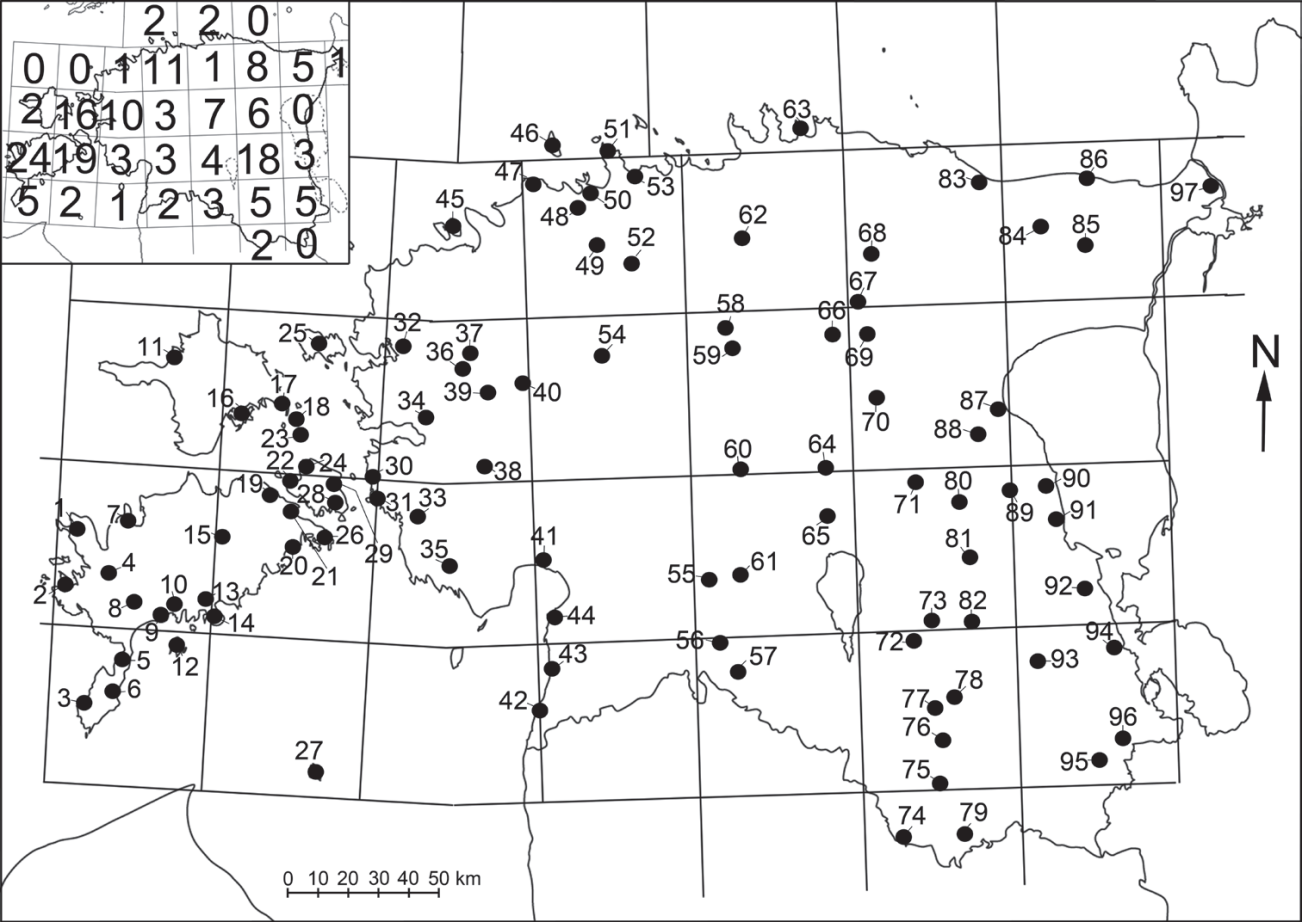
Collecting localities of Estonian Oniscidea and numbers of sampled localities per 50 × 50 km UTM squares. For further details, see Table 1.

As complete as possible, a bibliography of historical records of terrestrial isopods in Estonia was compiled. New material was collected using: (1) pitfall traps, (2) Tullgren funnels, (3) sifting moss, leaf litter, and detritus with standard entomological sieves, (4) manual searching in suitable habitats, and (5) as bycatch of non-target species with window traps (attached to tree trunks; Sammet et al. 2016) and Malaise traps (for details of the Estonian Malaise trap project, see Tomasson et al. 2014). The material was collected from 172 localities throughout Estonia (Table 1; Figure 1). All studied material is preserved in 80% ethanol and deposited in the entomological collection of Estonian University of Life Sciences (**IZBE**) and soil biology laboratory of TalTech Tartu College (**TTUSB**), both in Tartu, Estonia. Various keys for European woodlice were used for identification (Palmén 1946; Frankenberger 1959; Vandel 1960, 1962; Gruner 1966; Oliver and Meecham 1993). The distributions of Estonian species (Fig. 5) are presented in a 50 × 50 km UTM grid (compiled using Adobe Photoshop CS5 Extended). The images of the general habitus were combined using the LAS V.4.1.0 software from multiple gradually focused images of the specimens in alcohol taken by a Leica DFC 450 camera attached to Leica 205C stereomicroscope.

**Table 1.** Collecting localities of Estonian Oniscidea. The localities’ numbers correspond to those on Figure 1. Localities within a range of less than 10 km are presented by one number, the different place names (sub-localities) under one number are designated consecutive letters (the coordinates apply only to the first of them).

No.	Latitude, Longitude	Name	Methods used
1	58.3132°N, 21.9089°E	a Eeriksaare 1, b Eeriksaare 2, c Kõruse 1, d Kõruse 2, e Tammese, f Neeme	manual collecting; pitfall trapping; Tullgren funnel
2	58.3009°N, 21.9351°E	a Atla 1, b Atla 2, c Atla 3	manual collecting; pitfall trapping
3	57.9767°N, 21.9954°E	a Türju, b Sõrve south	manual collecting; pitfall trapping
4	58.2731°N, 22.0114°E	a Leedri, b Kipi, c Viidu-Mäebe, d Audaku, e Sutru, f Nakimetsa, g Pitkasoo	sifting soil and litter; manual collecting; pitfall trapping, Tullgren funnel
5	58.1213°N, 22.1966°E	Kaugatoma	manual collecting; pitfall trapping
6	58.0000°N, 22.1667°E	Viieristi	manual collecting
7	58.5167°N, 22.2167°E	a Mustjala, b Kugalepa, c Panga	manual collecting; Tullgren funnel
8	58.3186°N, 22.3057°E	a Mõnnuste, b Paadla	manual collecting; pitfall trapping
9	58.2425°N, 22.4249°E	Nasva	manual collecting; pitfall trapping
10	58.2496°N, 22.4800°E	Kuressaare	manual collecting; pitfall trapping
11	58.9445°N, 22.4361°E	a Paope b Reigi	manual collecting; pitfall trapping
12	58.1225°N, 22.5013°E	Abruka	manual collecting; Tullgren funnel
13	58.3005°N, 22.6337°E	Ilpla	manual collecting; pitfall trapping
14	58.2433°N, 22.6741°E	a Vanamõisa 1, b Vanamõisa 2, c Vanamõisa 3	manual collecting; pitfall trapping
15	58.4563°N, 22.7076°E	a Tika, b Võrsna	manual collecting; pitfall trapping; Tullgren funnel
16	58.7683°N, 22.8143°E	Kassari	manual collecting; pitfall trapping
17	58.8639°N, 22.9836°E	a Aruküla 1, b Aruküla 2, c Saarnaki, d Heltermaa, e Sarve	manual collecting; pitfall trapping; Tullgren funnel
18	58.7769°N, 23.0472°E	a Hanikatsi, b Langekare	manual collecting; pitfall trapping
19	58.5842°N, 23.0236°E	Orinõmme	manual collecting; pitfall trapping
20	58.4389°N, 23.0681°E	Asva 1	manual collecting; pitfall trapping
21	58.5557°N, 23.0879°E	Orissaare	manual collecting; pitfall trapping
22	58.6132°N, 23.0850°E	Koguva	manual collecting; pitfall trapping
23	58.7421°N, 23.1349°E	Ahelaid	Tullgren funnel
24	58.6412°N, 23.1536°E	a Paenase, b Pallasmaa, c Nõmmküla, d Üügu	manual collecting; pitfall trapping; Tullgren funnel
25	58.9917°N, 23.1928°E	Vormsi	manual collecting; pitfall trapping; Tullgren funnel
26	58.4617°N, 23.2111°E	a Kahtla, b Kübassaare	manual collecting; pitfall trapping Tullgren funnel
27	57.8006°N, 23.2283°E	a Ruhnu 1, b Ruhnu 2	manual collecting; window pane trap
28	58.5786°N, 23.2653°E	a Mäla 1, b Mäla 2, c Võiküla 3, d Võiküla 1	manual collecting; pitfall trapping
29	58.6501°N, 23.3133°E	a Lõetsa 1, b Lõetsa 2	manual collecting; pitfall trapping
30	58.6413°N, 23.5133°E	Hanila	manual collecting; pitfall trapping
31	58.5880°N, 23.5286°E	a Virtsu, b Puhtu, c Laelatu, d Pivarootsi	manual collecting; pitfall trapping; Tullgren funnel
32	59.0084°N, 23.6934°E	Linnamäe	manual collecting; pitfall trapping
33	58.5337°N, 23.8299°E	Paadermaa	manual collecting; pitfall trapping
34	58.8312°N, 23.8785°E	a Keskvere, b Patsu	manual collecting; pitfall trapping; Tullgren funnel
35	58.3818°N, 23.9810°E	a Ermistu, b Tõhela	manual collecting; pitfall trapping; Tullgren funnel
36	58.9020°N, 24.0287°E	a Marimetsa, b Kullamaa 1, c Kullamaa 2	manual collecting; Tullgren funnel
37	58.9947°N, 24.0559°E	Risti	manual collecting; Tullgren funnel
38	58.6455°N, 24.1253°E	Kurese	manual collecting; Tullgren funnel
39	58.7760°N, 24.2496°E	Vigala	manual collecting; Tullgren funnel
40	58.8960°N, 24.3758°E	Sõtke	manual collecting; pitfall trapping; Tullgren funnel
41	58.3864°N, 24.3695°E	a Valgeranna, b Pärnu	manual collecting; Tullgren funnel
42	58.0195°N, 24.4532°E	Kabli	manual collecting; Tullgren funnel
43	58.0807°N, 24.4889°E	a Häädemeeste, b Palitsa	manual collecting; Tullgren funnel
44	58.2429°N, 24.4965°E	Tahkuranna	manual collecting; Tullgren funnel
45	59.3339°N, 23.9703°E	Väike-Pakri	manual collecting; Tullgren funnel
46	59.5933°N, 24.5025°E	Naissaar	manual collecting; Tullgren funnel
47	59.4425°N, 24.5228°E	a Rannamõisa MKA b Muraste c Tõmmiku	manual collecting; pitfall trapping; Tullgren funnel
48	59.3411°N, 24.6386°E	a Tänassilma cave, b Vana-Mustamäe	manual collecting; pitfall trapping; Tullgren funnel
49	59.2661°N, 24.6483°E	Kasemetsa	manual collecting; Tullgren funnel
50	59.4293°N, 24.7777°E	central Tallinn	manual collecting; Tullgren funnel
51	59.5297°N, 24.8578°E	Lubja	manual collecting; Tullgren funnel
52	59.2378°N, 24.9311°E	Sõmeru	manual collecting; Tullgren funnel
53	59.4631°N, 24.9378°E	a Maardu, b Muuga, c Ülgase cave	manual collecting; pitfall trapping; Tullgren funnel
54	58.9703°N, 24.7294°E	a Kuusiku, b Raela, c Raikküla	manual collecting; pitfall trapping; Tullgren funnel
55	58.3333°N, 25.3000°E	Kõpu	manual collecting; Tullgren funnel
56	58.1562°N, 25.3390°E	Koodioru	manual collecting
57	58.1557°N, 25.4360°E	a Halliste, b Viivre	manual collecting; pitfall trapping; Tullgren funnel
58	59.0835°N, 25.4053°E	Mustla	manual collecting; Tullgren funnel
59	58.8893°N, 25.5725°E	Paide	manual collecting; Tullgren funnel
60	58.6304°N, 25.6196°E	a Koksvere, b Kirivere, c Kõo	manual collecting; Tullgren funnel
61	58.3738°N, 25.6127°E	Viljandi	manual collecting; Tullgren funnel
62	59.2553°N, 25.6669°E	Aegviidu	manual collecting; Tullgren funnel
63	59.6049°N, 25.9230°E	a Käsmu, b Natturi	manual collecting; pitfall trapping; Tullgren funnel
64	58.6548°N, 25.9685°E	Põltsamaa	manual collecting; Tullgren funnel
65	58.5332°N, 25.9468°E	a Kolga-Jaani, b Lalsi	manual collecting; Tullgren funnel
66	58.9996°N, 26.1168°E	Liigvalla	manual collecting; Tullgren funnel
67	59.0970°N, 26.1826°E	Vao	manual collecting; Tullgren funnel
68	59.2742°N, 26.1955°E	a Lasila, b Karunga, c Levala	manual collecting; pitfall trapping; Tullgren funnel
69	59.0233°N, 26.2444°E	Kamariku	manual collecting; pitfall trapping; Tullgren funnel
70	58.8669°N, 26.2625°E	a Tooma, b Kärde	manual collecting; pitfall trapping; Tullgren funnel
71	58.5948°N, 26.3631°E	a Kursi, b Tõrve, c Altnurga	manual collecting; Tullgren funnel
72	58.1072°N, 26.2767°E	Atra	manual collecting; pitfall trapping; Tullgren funnel
73	58.2066°N, 26.3825°E	a Käärdi, b Peedu	manual collecting; pitfall trapping; Tullgren funnel
74	57.5962°N, 26.2855°E	Olina	manual collecting; Tullgren funnel
75	57.7193°N, 26.5000°E	Mähkli	manual collecting; Tullgren funnel
76	57.8611°N, 26.5241°E	Vana-Antsla	manual collecting; Tullgren funnel
77	57.9510°N, 26.4368°E	Ilmjärve	manual collecting; Tullgren funnel
78	58.0062°N, 26.6073°E	Kaagvere	manual collecting; Tullgren funnel
79	57.5727°N, 26.6413°E	Mõisamõtsa	manual collecting; Tullgren funnel; window pane trap
80	58.5604°N, 26.6285°E	Valgma	manual collecting; Tullgren funnel
81	58.4103°N, 26.6394°E	a Tiksoja, b Tähtvere bog, c Õssu, d Maramaa, e TartuTähtvere, f Tartu central, g Tartu Aardla, h Raadi, I Aruküla cave	manual collecting; pitfall trapping; Tullgren funnel
82	58.2302°N, 26.7011°E	Kambja	manual collecting; Tullgren funnel
83	58.5950°N, 26.7719°E	a Tüükri, b Kalvi, c Oru, d Aseri	manual collecting; pitfall trapping; Tullgren funnel
84	59.3019°N, 26.8818°E	a Ilmaste, b Nüri, c Aidu	manual collecting; pitfall trapping; Tullgren funnel
85	59.2289°N, 27.3247°E	Mäetaguse NR	window pane trap
86	59.4448°N, 27.3348°E	Valaste	manual collecting; Tullgren funnel
87	58.7841°N, 26.9330°E	a Nõmme b Ruskavere	manual collecting; pitfall trapping; Tullgren funnel
88	58.7279°N, 26.8260°E	Odivere	manual collecting; pitfall trapping; Tullgren funnel
89	58.5170°N, 26.9224°E	a Välgi b Pataste	manual collecting; pitfall trapping; Tullgren funnel
90	58.6033°N, 27.1304°E	Alatskivi	manual collecting; Tullgren funnel
91	58.4968°N, 27.2376°E	Varnja	manual collecting; Tullgren funnel
92	58.2750°N, 27.3250°E	Järvselja	manual collecting; sifting soil and litter; Tullgren funnel
93	58.1148°N, 27.0474°E	Saessaare	manual collecting; Tullgren funnel
94	58.0965°N, 27.4744°E	Ristipalo	manual collecting; Tullgren funnel
95	57.7447°N, 27.3335°E	a Möldri b Parmu	manual collecting; Tullgren funnel
96	57.8433°N, 27.4655°E	Piusa	manual collecting; Tullgren funnel
97	59.3573°N, 28.1970°E	Narva	manual collecting; Tullgren funnel

## Results

Altogether 14142 specimens were collected. Of these, 10915 were identified to the species level. The following list contains all the known published records of Estonian woodlice, followed by numbers of studied specimens and collecting localities. Full details for each record from each locality are given in Suppl. material 1. An asterisk (*) indicates a species new to Estonia. The full list of records with all details will be available through the Estonian eBiodiversity portal (http://elurikkus.ut.ee; Abarenkov et al. 2010) and Global Biodiverdsity Information Facility (https://www.gbif.org). Nomenclature and synonymics follow Schmalfuss (2003).

### Ligiidae

***Ligidiumhypnorum* Cuvier, 1792**

Figs 4B, 5A

**Published sources.**Herold 1930: 478–479; Vilbaste and Vilbaste 1993: 317.

**Studied material.** 117 specimens from 13 localities (loc. 30a, 47b, 47c, 54c, 57b, 83c, 87a, 87b, 88a, 89a, 92a, 95a, 95b).

**Comments.** A locally abundant species, with no records from Estonian islands. It has been described as widespread in Estonia also in the past (Herold 1930). The findings are from different habitats: fresh to mesic forests, meadows, arable fields and gardens. Present also in Lithuania (Vilisics et al. 2012) and Latvia (Spuņģis 2008) but not Finland (Boxshall G 2013).

### Trichoniscidae

***Trichoniscuspusillus* Brandt, 1833**

Figs 3A, 5B

**Published sources.**Herold 1927: 6; Herold 1928: 215; Herold 1930: 479 (as *T.elisabethae* Herold, 1923; *T.elisabethaevar.estoniensis* Herold, 1927; *T.caelebs* Verhoeff, 1917); Vilbste 1970: 170 (as *T.pusilluscaelebs* Vh.); Vilbaste et al. 1985: 151 (as *T.pusilluscaelebs* Vh.); Remm 1988: 127 (as *T.pusilluscaelebs* Vh.); Vilbaste and Vilbaste 1993: 317.

**Studied material.** 117 specimens from 24 localities (loc. 1e, 4d, 17a, 23b, 25a, 34a, 35a, 39a, 40c, 47a, 50a, 51a, 57a, 58c, 68a, 74a, 78a, 78b, 78e, 80a, 80d, 83a, 89b, 92a).

**Comments.** Once reported as the most common species of Trichoniscidae (e.g. Herold 1927, 1930), the species appears to have become less abundant. It is widespread in various habitats (bogs, different types of forests, meadows, and urban areas), but is more common in moist habitats and is often associated with decaying wood. The species is known to be mainly parthenogenetic (Gruner 1966; De Smedt et al 2016), and the collected material consisted only of female specimens. Thus, no male characters were available for study and it cannot be ruled out that some specimens were misidentified and other *Trichoniscus* species may also be present in Estonia as very rare (e.g. *T.provisorius* or *T.pygmaeus*). The taxonomic status of the described varieties *T.elisabethae* Herold, 1923 and T.elisabethaevar.estoniensis Herold, 1927 is unclear, but we follow the Schmalfuss (2003) catalogue and treat them as *T.pusillus*. Present also in Latvia (Spuņģis 2008) and Finland (Palmén 1946; Vilisics and Terhivuo 2009).

* ***Hyloniscusriparius* (C. Koch, 1838)**

Figs 3B, 5C

**Studied material.** 202 specimens from 22 localities (loc. 12a, 23b, 35b, 39a, 39a, 49a, 50a, 52b, 53a, 55b, 57a, 58b, 59a, 60b, 61a, 69c, 70b, 78a, 78e, 78g, 83a, 85a).

**Comments.** The species is widespread and common, but has only recent records and is probably extending its range in the Europe. It has been often found in human settlements, but also seashore habitats and different types of forests, except the very dry ones. Present also in Latvia (Spuņģis 2008) and Finland (Vilisics and Terhivuo 2009).

***Haplophthalmusmengii* (Zaddach, 1844)**

Fig. 5D

**Published source.**Herold 1930: 479–480.

**Comments.** Reported as rare, with only one finding locality in northern Estonia (Herold 1930). No recent records. Present also in Latvia (Spuņģis 2008) and Finland (Palmén 1946).

### Platyarthridae

* ***Platyarthrushoffmannseggii* Brandt, 1833**

Figs 4A, 5E

**Studied material.** 3 specimens from 1 locality (loc. 80g).

**Comments.** A myrmecophilous species, found from a nest of *Lasiusniger* (Linnaeus, 1758). There are no records from the northern Baltic region so far, but it has recently been found in Lithuania (Šatkauskienė 2017), and a population has also been found in Finland (Lehtinen 1961). However, due to the destruction of the only known locality, the species could be extinct there now (Vilisics and Terhivuo 2009).

### Trachelipodidae

***Trachelipusrathkii* (Brandt, 1833)**

Figs 2A, 5F

**Published sources.**Herold 1927: 52 (as *Porcelliorathkei*); Herold 1930: 476 (as *Tracheoniscusrathkei* (Brandt, 1833)); Vilbaste 1970: 170 (as *Tracheoniscusrathkei* (Brandt, 1833)); Vilbaste et al. 1985: 151 (as *Tracheoniscusrathkei* (Br.)).

**Studied material.** 3180 specimens from 114 localities (loc. 1a, 1c, 1e, 1f, 3a, 3b, 4c, 6a, 7a, 9a, 9b, 10a, 10b, 11a, 12a, 13a, 14b, 15a, 15b, 16a, 17a, 17b, 17c, 17d, 18a, 18b, 18c, 19a, 20a, 21a, 22a, 23a, 23b, 23c, 25a, 27a, 28a, 28b, 29a, 30a, 30c, 30d, 33a, 33b, 34a, 34b, 35b, 35b, 35c, 35c, 37a, 38a, 39a, 40a, 40b, 41a, 41b, 42a, 46b, 51a, 51b, 52a, 52b, 53a, 55a, 56a, 57a, 58a, 58b, 59a, 60b, 61a, 62a, 63b, 64a, 65a, 66a, 66b, 66c, 66c, 67a, 68b, 69a, 69b, 70a, 71a, 72a, 73a, 76a, 77a, 78a, 78d, 78e, 78f, 78f, 79a, 80a, 80b, 80c, 80d, 81a, 81b, 81c, 82a, 83a, 84a, 85a, 86a, 87a, 90a, 91a, 91b).

**Comments.** One of the most common species in Estonia, in all kinds of habitats (both anthropogenic and natural, except bogs). It has also been described as widespread and common in Estonia in the past (Herold 1927, 1930). Present also in Leningrad region (European Russia, Kuznetsova and Gongalsky 2012), Latvia (Spuņģis 2008), and Finland (Palmén 1946).

### Porcellionidae

***Porcellioscaber* Latreille, 1804**

Figs 2B, 5G

**Published sources.**Herold 1927: 52; Herold 1930: 481; Vilbaste et al. 1985: 151.

**Studied material.** 217 specimens from 13 localities (loc. 3b, 24a, 26a, 26b, 30b, 35c, 46b, 48a, 63a, 78c, 78e, 78f, 84a).

**Comments.** The species was described as purely synanthropic in continental Estonia and free-living in western Estonian islands (Herold 1927, 1930). The studied material contains findings from and outside of human settlements (including different forests, grasslands, and seashore) both from western islands and continent. Present also in Novgorod region (European Russia, Kuznetsova and Gongalsky 2012), Latvia (Spuņģis 2008), and Finland (Palmén 1946).

***Porcelliospinicornis* Say, 1818**

Figs 2C, 5H

**Published sources.**Herold 1927: 52; Herold 1930: 481 (as *P.pictus* Brandt, 1833); Vilbaste et al. 1985: 151 (as *P.pictus* Br.).)

**Studied material.** 68 specimens from 19 localities (loc. 4b, 6b, 20b, 32a, 35b, 39a, 40b, 46a, 51c, 52b, 60a, 61a, 78f, 78h, 78i, 84a, 89a, 93a, 97).

**Comments.** A common and widespread species, often found on stone walls in human settlements, but also in mesic deciduous forests. Present also in Leningrad region (European Russia, Kuznetsova and Gongalsky 2012), Latvia (Spuņģis 2008), and Finland (Palmén 1946).

***Porcellionidespruinosus* (Brandt, 1833)**

**Published source.**Herold 1930: 476.

**Comments.** No recent records. This species has been described as purely synanthropic in Estonia (Herold 1930). Present also in Latvia (Spuņģis 2008) and Finland (Palmén 1946).

### Cylisticidae

***Cylisticusconvexus* (De Geer, 1778)**

Figs 2D, 5I

**Published sources.**Herold 1927: 51; 480 Herold 1930: 480.

**Studied material.** 825 specimens from 5 localities (loc. 46b, 66a, 78e, 78f, 78g).

**Comments.** This species is widespread and locally quite abundant, both in human settlements and in forests, under stones or in rotten logs. It has been described as widespread and mainly synanthropic in Estonia by W. Herold, with free-living populations in northern and western Estonian islands (Herold 1930). Present also in Latvia (Spuņģis 2008) and Finland (Palmén 1946).

### Oniscidae

***Oniscusasellus* Linnaeus, 1758**

Figs 2E, 5J

**Published sources.**Fischer 1778: 167 (questionable; see comment in Introduction); Herold 1930: 480.

**Studied material.** 433 specimens from 5 localities (loc. 4b, 6, 36b, 47b, 48b).

**Comments.** The species seems to be free-living on the island of Saaremaa, but synanthropic and sometimes quite abundant elsewhere. Herold (1930) described it as being widespread but purely synanthropic in Estonia. Present also in Latvia (Spuņģis 2008), Pskov region (European Russia, Kuznetsova and Gongalsky 2012), and Finland (Palmén 1946).

### Armadillidiidae

***Armadillidiumopacum* (Koch, 1841)**

Figs 3D, 5K

**Published sources.**Herold 1927: 53; Herold 1930: 483–485; Vilbaste et al. 1985: 151.

**Figure 2.**
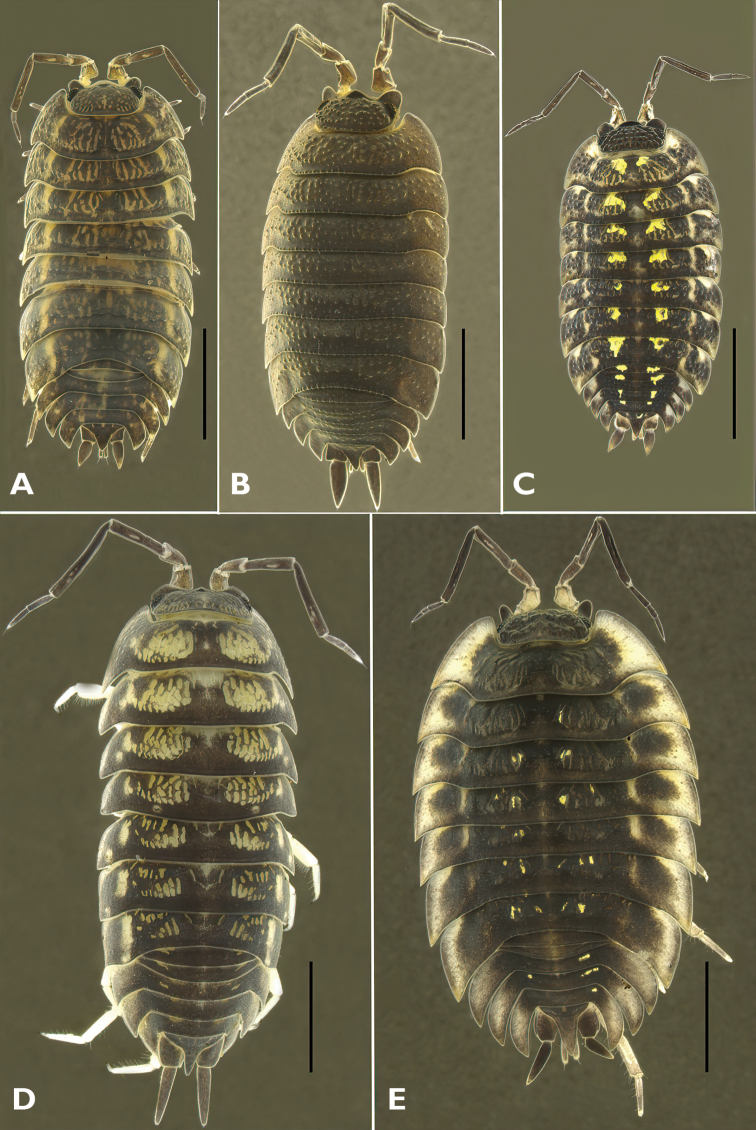
Habitus of Estonian Oniscidea species **A***Trachelipusrathkii***B***Porcellioscaber***C***Porcelliospinicornis***D***Cylisticusconvexus***E***Oniscusasellus*. Scale bars: 2 mm.

**Figure 3.**
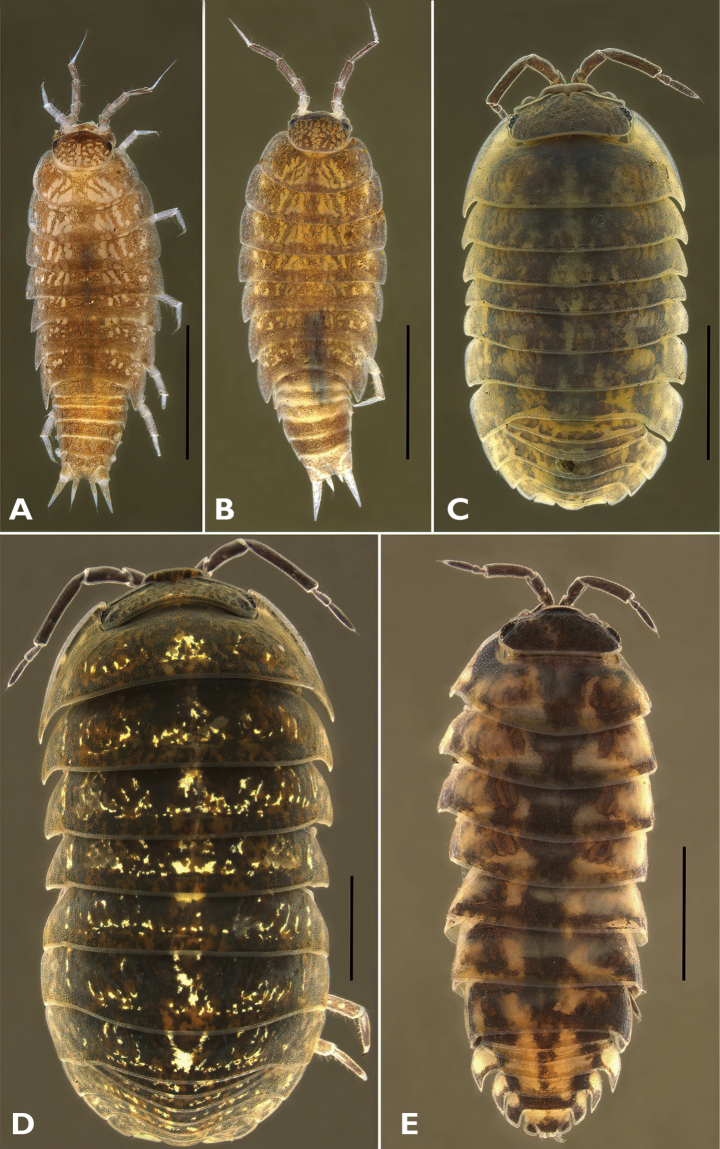
Habitus of Estonian Oniscidea species **A***Trichoniscuspusillus***B***Hyloniscusriparius***C***Armadillidiumzenckeri***D***Armadillidiumopacum***E***Armadillidiumpulchellum*. Scale bars: 1 mm.

**Figure 4.**
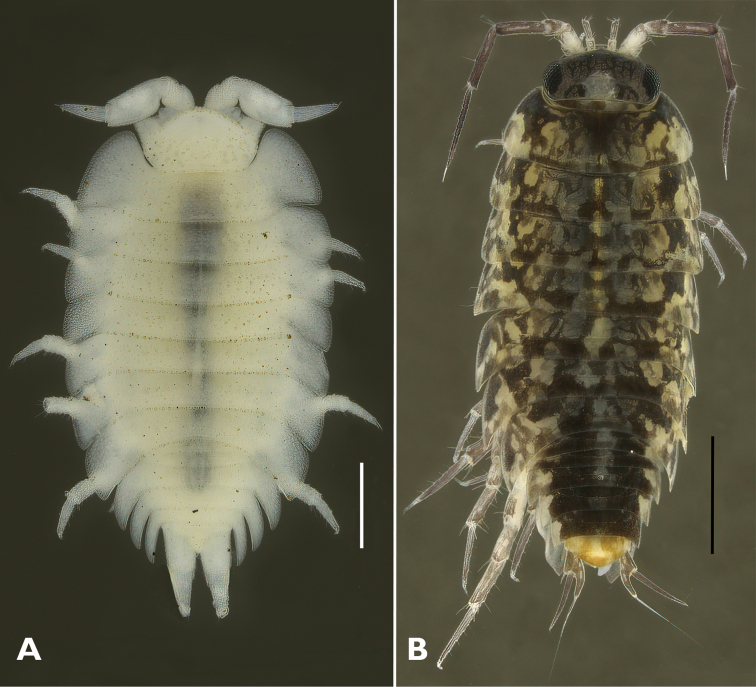
Habitus of Estonian Oniscidea species **A***Platyarthrushoffmannseggii***B***Ligidiumhypnorum*. Scale bars: 0.5 mm (**A**); 1 mm (**B**).

**Studied material.** 5294 specimens from 44 localities (loc. 1a, 1b, 1c, 1d, 1e, 1f, 2a, 2b, 2c, 4b, 5a, 6a, 8a, 8c, 9a, 9b, 11a, 13a, 14a, 14b, 14c, 14c, 15b, 16a, 17a, 17b, 17c, 17d, 18b, 19a, 20a, 21a, 23a, 23c, 25a, 25b, 27a, 28a, 28b, 29a, 30a, 30d, 31a, 35a, 37a).

**Comments.** Very common in western Estonia and islands (in forests, grasslands, and coastal habitats) but rare elsewhere. Present also in Latvia (Spuņģis 2008) and Finland (Palmén 1946).

***Armadillidiumpictum* Brandt, 1833**

Fig. 5L

**Published sources.**Herold 1927: 53; Herold 1930: 482–483.

**Studied material.** 5 specimens from 3 localities (loc. 28a, 44a, 45a).

**Comments.** A rare species found only in northern Estonia and Muhu island in coastal habitats (broad-leaved forest under limestone escarpment, pine forest near seashore, alvar grassland). Present also in Latvia (Spuņģis 2008) and Finland (Palmén 1946).

**Figure 5.**
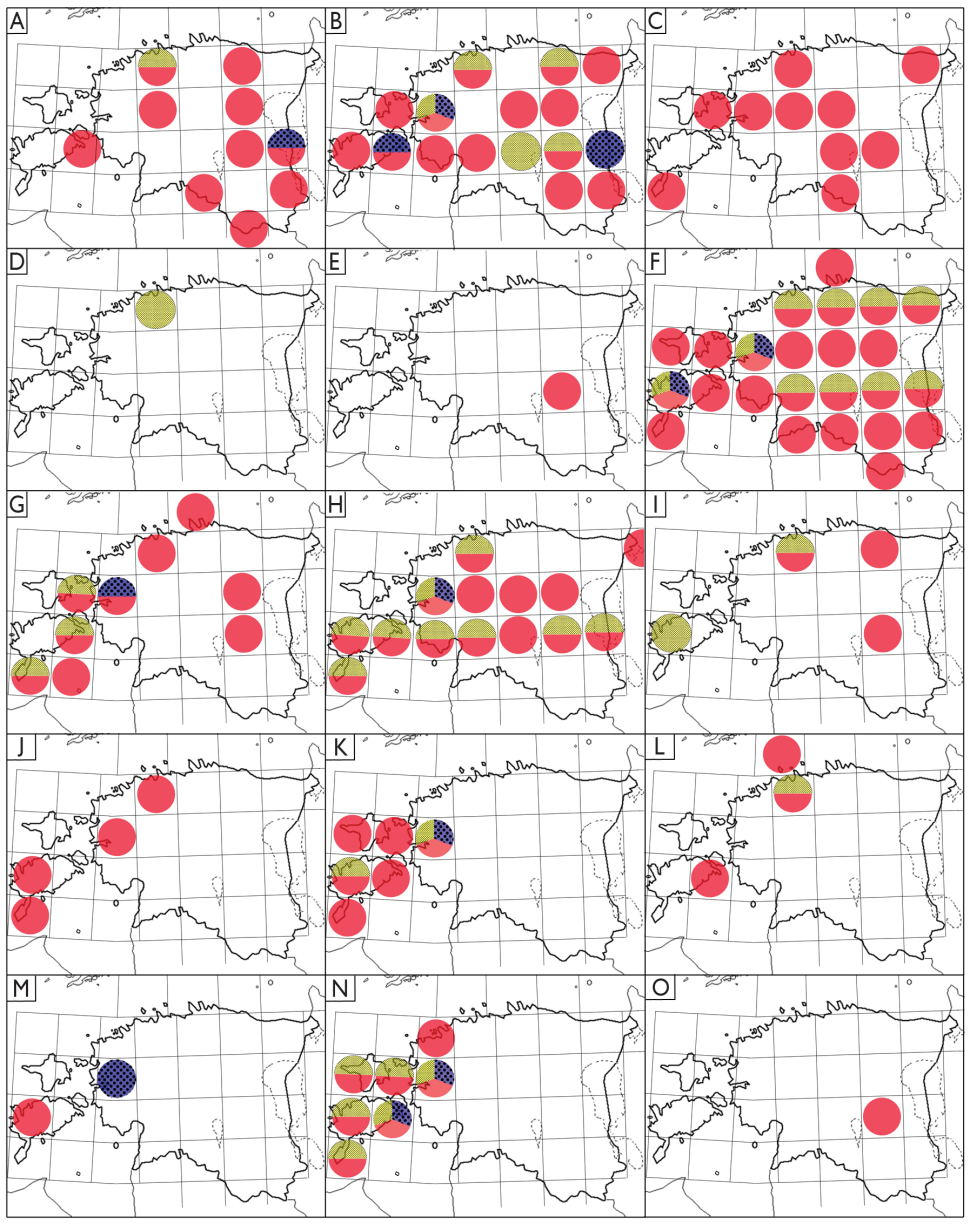
Distribution of Estonian Oniscidea. Red denotes studied specimens (1984–2020), blue = literature data 1970–1993, yellow = literature data 1927–1930 **A***Ligidiumhypnorum***B***Trichoniscuspusillus***C***Hyloniscusriparius***D***Haplophthalmusmengii***E***Platyarthrushoffmannseggii***F***Trachelipusrathkii***G***Porcellioscaber***H***Porcelliospinicornis***I***Cylisticusconvexus***J***Oniscusasellus***K***Armadillidiumopacum***L***Armadillidiumpictum***M***Armadillidiumpulchellum***N***Armadillidiumzenckeri***O***Armadillidiumvulgare*. *Porcellionidespruinosus* has been omitted since the published source mentions no specific localities.

***Armadillidiumpulchellum* (Zenker, 1798)**

Figs 3E, 5M

**Published sources.**Herold 1930: 481–482; Vilbaste et al. 1985: 151.

**Studied material.** 1 specimen from 1 locality (loc. 4d).

**Comments.** A rare species found only on Saaremaa island in western Estonia (in a spring fen). Present also in Latvia (Spuņģis 2008) and Finland (Palmén 1946).

***Armadillidiumvulgare* (Linnaeus, 1758)**

Fig. 5O

**Published source.**Chinery 2005: 300.

**Studied material.** 1 specimen from 1 locality (loc. 81a).

**Comments.** A rare synanthropic species with only one finding from Estonia (from suburban area in Tartu). Present also in Latvia (Spuņģis 2008) and Finland (Palmén 1946).

***Armadillidiumzenckeri* Brandt, 1833**

Figs 3C, 5N

**Published sources.**Herold 1927: 53; Herold 1930: 485–490; Vilbaste 1970: 170; Vilbaste et al. 1985: 151.

**Studied material.** 452 specimens from 44 localities (loc. 1a, 1b, 1d, 1e, 1f, 2a, 2b, 2c, 3a, 4a, 4c, 4e, 5a, 7a, 8b, 9a, 9b, 11a, 13a, 14a, 14b, 15b, 16a, 17a, 17b, 17c, 17d, 19a, 20a, 21a, 23a, 23b, 23c, 25a, 28a, 28b, 29a, 30a, 30d, 36a, 37a, 37a, 37a, 43a).

**Comments.** Common in western Estonia and islands, but rare elsewhere. Present in dry to mesic forests and different grasslands. Present also in Latvia (Spuņģis 2008) and Finland (Palmén 1946).

## Discussion

There are reliable records of 16 species of terrestrial isopods from Estonia. One species has been dubiously claimed to occur in Estonia, and it is presently not included in the checklist. We failed to find any records or specimens to support the occurrence of *Philosciamuscorum* (Scopoli, 1763) in Estonia, although marked as “present” in Fauna Europaea database (Boxshall G 2013). The species is, however, present in the neighbouring Latvia (Spuņģis 2008), and its occurrence in Estonia is not impossible. Two species, viz. *Haplophthalmusmengii* (Zaddach, 1844) and *Porcellionidespruinosus* (Brandt, 1833), have not been recently collected and are included here based on literature records only. The fauna is very similar to neighbouring Latvia and southern Finland, with which all species shared, except for *Ligidiumhypnorum* and *P.hoffmannseggii*, but the apparent absence of the latter in Latvia can be possibly explained by its rarity and lack of studies in its specific habitat (ant nests). Comparing the recent records with older ones, it seems that the distribution and abundance of some species have remained approximately the same over the past century, whereas some other species appear to have become rarer or have expanded their ranges. *Porcellioscaber* was reported as a synanthropic species in continental Estonia (Herold 1930), but we found it also in the field there. The same applies to *Oniscusasellus*. The existing Baltic records of *Hyloniscusriparius* are from Lithuania and southern Latvia (Spuņģis 2008; Tuf et al. 2014). The first Estonian records are from 2015, and given that the species was found during the 2003–2007 studies only in south-western Latvia (Spuņģis 2008), its range may have shifted remarkably quickly (by more than 300 km northwards in only a decade). An expansion of the species range northwards and eastwards has also been detected in European Russia in recent decades (Gongalsky et al. 2013) and has recently reached also the Russian Far East (Gongalsky and Kuznetsova 2021). The first Finnish record of the species was from a greenhouse in 1946, but the first finding outside dates from 2007 (Vilisics and Terhivuo 2009). Several species are only found or are more common in western and northern Estonia, characterized by milder maritime climate and calcareous soil (*Armadillidiumopacum*, *A.pictum*, *A.pulchellum*, *A.zenckeri*, *Haplophthalmusmengii*). Seven species are known from areas neighbouring Estonia and may have been not collected due to rarity or very local distribution: *Porcelliumconspersum* (C.Koch, 1841), *Philosciamuscorum* (Scopoli, 1763), *Haplophthalmusdanicus* Budde-Lund, 1879, *Porcelliodilatatus* Brandt, *Porcelliolaevis* Latreille, 1804, and *Armadillidiumnasatum* Budde-Lund 1885. The range of *Trichoniscusprovisorius* Racovitza, 1908 reaches Poland (Jędryczkowski 1979), and there are other widespread *Trichoniscus* species, e.g. *T.alemannicus* or *T.pygmaeus* Sars 1898, in central Europe (the latter reaching southern Russia in the east; Kuznetsova and Gongalsky 2012), but due to lack of male specimens these species may remain as yet undetected in the Baltic countries. Introduced species can sometimes be found in greenhouses and may be expected to be found in the future too; several of the 25 species found in Finland (Vilisics and Terhivuo 2009) are found only indoors. It seems probable that the number of naturally occurring species might be closer to 19 as in neighbouring Latvia (Spuņģis 2008).

## Supplementary materials

Supplementary material 1 Supplementary data Data type occurences Explanation note Finding details of studied Estonian Oniscidea specimens from each locality (repeated findings from exactly the same locality are omitted). https://binary.pensoft.net/file/604083This dataset is made available under the Open Database License (http://opendatacommons.org/licenses/odbl/1.0/). The Open Database License (ODbL) is a license agreement intended to allow users to freely share, modify, and use this Dataset while maintaining this same freedom for others, provided that the original source and author(s) are credited.Kaarel Sammet

## References

[B1] Abarenkov K Tedersoo L Nilsson RH Vellak K Saar I Veldre V Parmasto E Prous M Aan A Ots M Kurina O Ostonen I Jõgeva J Halapuu S Põldmaa K Toots M Truu J Larsson K Kõljalg U (2010) PlutoF – a web based workbench for ecological and taxonomic research, with an online implementation for fungal ITS sequences. Evolutionary Bioinformatics 6: 189–196. 10.4137/EBO.S6271

[B2] Boxshall G (2013) Fauna Europaea: Isopoda, Philosciidae. Fauna Europaea version 2017.06. https://fauna-eu.org [accessed 10.V.2020]

[B3] Fischer JB (1778) Versuch einer Naturgeschichte von Livland. Johann Gottlob Immanuel Breitkopf, Leipzig, 415 pp.

[B4] Chinery M (2005) Euroopa putukad. Eesti Entsüklopeediakirjastus, 320 pp. [Insects of Britain and Northern Europe; in Estonian]

[B5] De Smedt P Arijs G Boeraeve P Proesmans W (2016) *Trichoniscusalemannicus* Verhoeff, 1917 a new species of woodlouse for Belgium (Isopoda: Trichoniscidae). Bulletin de la Société royale belge d’Entomologie 152: 104–108.

[B6] Frankenberger Z (1959) Fauna ČSR, Svazek 14. Stejnonožci suchozemští – Oniscoidea. Prague, 212 pp. [Fauna of the ČSR, vol. 14; in Chech]

[B7] Gongalsky KB Kuznetsova DM Filimonova ZhV Shakhab SV (2013) Distribution and ecology of the invasive species of woodlice *Hyloniscusriparius* (C. Koch, 1838) (Isopoda, Oniscidea, Trichoniscidae) in Russia. Russian Journal of Biological Invasions 4(2): 116–119. 10.1134/S2075111713020045

[B8] Gongalsky KB Kuznetsova DM (2021) Distribution of alien species of woodlice (Crustacea, Isopoda, Oniscidea) in the Russian Far East. Russian Journal of Biological Invasions 12(1): 44–49. 10.1134/S2075111721010069

[B9] Gruner HE (1966) Die Tierwelt Deutschlands. 53. Teil. Krebstiere oder Crustacea. V. Isopoda, 2. Lieferung. Gustav Fischer, Jena, 151–380.

[B10] Herold W (1927) Land-Isopoden aus dem Ostbaltikum. Zoologischer Anzeiger 72(1/2): 49–54.

[B11] Herold W (1928) Beiträge zur Kenntnis der Trichonisciden I. Die Untergattung *Spiloniscus* Racovitza in Deutschland und im Ostbaltikum. Zoologische Jahrbücher, Abteilung für Systematik, Ökologie und Geographie der Tiere 57: 215–252.

[B12] Herold W (1930) Beiträge zur Verbreitung und Ökologie der Landisopoden des Ostbaltikums. Zeitschrift für Morphologie und Ökologie der Tiere 18(3): 474–53.

[B13] Jędryczkowski W (1979) Synantropijne równonogi lądowe (Isopoda, Oniscoidea) Polski. Fragmenta Faunistica 25(7): 95–105. [Synanthropic woodlice (Isopoda, Oniscoidea) of Poland; in Polish]

[B14] Kuznetsova DM Gongalsky KB (2012) Cartographic analysis of woodlice fauna of the former USSR. ZooKeys 176: 1–11. 10.3897/zookeys.176.2372 PMC3335401 22536095

[B15] Lehtinen PT (1961) *Platyarthrushoffmanseggi* Brandt (Isopoda) and *Blaniulusguttulatus* Bosc. (Diplopoda) found in the open in southwestern Finland. Archivum Societatis Zoologicae Botanicae Fennicae “Vanamo” 15(1–2): 106–109.

[B16] Oliver PG Meecham CJ (1993) Synopses of the British Fauna (N. S.) 49. Woodlice. Backhuys Publishers, London, 136 pp.

[B17] Palmén E (1946) Die Landisopoden Finnlands. Annales Zoologici Societatis Zoologicae Botanicae Fennicae “Vanamo” 11: 1–36.

[B18] Remm K (1988) Koljaku-Oandu reservaadi samblarinde loomastik suve teisel poolel. In: Etverk I (Ed.) Lahemaa Uurimused III.Rahvuspargi looduse inventeerimine. Valgus, Tallinn, 120–142. [Mesofauna of the moss layer of the Koljaku-Oandu Nature Reserve in the second half of summer; in Estonian]

[B19] Sammet K Talvi T Süda I Kurina O (2016) Pseudoscorpions (Arachnida: Pseudoscorpiones) in Estonia: new records and an annotated checklist. Entomologica Fennica 27(4): 149–163. 10.33338/ef.60259

[B20] Šatkauskienė I (2017) New record and additional data of terrestrial isopods in Kaunas region, Lithuania. Bulletin of the Lithuanian entomological society 1(29): 129–131.

[B21] Schmalfuss H (2003) World catalog of terrestrial isopods (Isopoda: Oniscidea). Stuttgarter Beiträge zur Naturkunde, Serie A, 654: 1–341.

[B22] Spuņģis V (2008) Fauna, distribution, habitat preference and abundance of the woodlice (Oniscidea) in Latvia. Latvijas Entomologs 45: 25–37.

[B23] Tomasson K Tammaru T Kurina O (2014) Harvestmen (Arachnida: Opiliones) in Estonia: results of the Estonian Malaise Trap Project. Entomologica Fennica 25(3): 142–156. 10.33338/ef.48267

[B24] Tuf IH Ivinskis P Rimsaite J (2014) Four terrestrial isopod species (Isopoda: Oniscidea) new for Lithuanian fauna and data on distribution of another seven species. New and Rare for Lithuania Insect Species 26: 86–89.

[B25] Vandel A (1960) Isopodes Terrestres (première partie). Faune de France 64. Lechevalier, Paris, 416 pp.

[B26] Vandel A (1962) Isopodes Terrestres (deuxième partie) Faune de France 66. Lechevalier, Paris, 515 pp.

[B27] Vilbaste J (1970) Koorikloomad (Crustacea). In: Haberman H Kaar E Kumari E (Eds) Lääne-Eesti rannikualade loodus.Valgus, Tallinn, 169–170. [Crustaceans (Crustacea); in Estonian]

[B28] Vilbaste J Haberman H Krall E Maavara V Martin A Remm E Remm H Siitan V Viidalepp J Vilbaste A (1985) Matsalu märgala maismaaselgrootud. In: Kumari E (Ed.) Matsalu – rahvusvahelise tähtsusega märgala.Valgus, Tallinn, 140–198. [Terrestial invertebrates of the Matsalu wetland; in Estonian]

[B29] Vilbaste J Vilbaste A (1993) Järvselja looduskaitsekvartali selgrootutest. Loodusuurijate Seltsi Aastaraamat 74: 304–330. [About the invertebrates of the Järvselja Forest Reserve; in Estonian]

[B30] Vilisics F Terhivuo J (2009) Inspection on materials contributing to the knowledge of terrestrial Isopoda (Crustacea, Oniscidea) in Finland. Memoranda Societatis pro Fauna et Flora Fennica 85(1): 9–15.

[B31] Vilisics F Ivinskis P Rimšaitė J (2012) Terrestrial isopods (Crustacea, Oniscidea) at the Baltic Sea coast in Lithuania. Zoology and Ecology 22(3): 1–7. 10.1080/21658005.2012.748517

